# Understanding challenges to malaria elimination in Nepal: a qualitative study with an embedded capacity-building exercise

**DOI:** 10.1186/s12936-019-3081-7

**Published:** 2019-12-21

**Authors:** Shiva Raj Adhikari, Vishnu P. Sapkota, Arjun K. Thapa, Yubraj Acharya

**Affiliations:** 1 Central Department of Economics, Tribhuvan University, Kirtipur, Nepal; 2 Institute of Medicine, Tribhuvan University, Kirtipur, Nepal; 3 School of Development and Social Engineering, Pokhara University, Pokhara, Nepal; 4 College of Health and Human Development, The Pennsylvania State University, 601L Ford Building, University Park, PA 16802 USA

**Keywords:** Nepal, Malaria, Elimination, Health systems

## Abstract

**Background:**

The Nepalese Government has made significant progress toward the elimination of malaria. However, given the surge in the prevalence of non-communicable diseases, such as diabetes and hypertension, and the localized nature of malaria prevalence, malaria elimination will remain a challenge. In the current study, the authors sought to understand local perceptions on threats to malaria elimination in three endemic districts.

**Methods:**

The authors conducted a capacity-building exercise embedded within a qualitative study. The study component aimed to understand how local policymakers and actors perceive challenges in malaria elimination. For them to be able to articulate the challenges, however, an understanding of malaria elimination in the context of a broader health system in Nepal would be required. The capacity-building component, thus, involved providing that knowledge.

**Results:**

Although the prevalence of malaria is high in the three districts where the study was conducted, there are significant gaps in human resources, diagnosis and treatment, and the provision of indoor residual spraying and long-lasting insecticide treated nets. More importantly, the authors’ experience suggests that it may be possible to capitalize on local expertise in order to identify gaps in malaria elimination at a sub-national level by building in a capacity-building exercise within a study.

**Conclusions:**

Locals in three malaria-endemic districts of Nepal perceive that there are significant gaps in human resources, diagnosis and treatment, the provision of insecticide treated nets, and indoor residual spraying.

## Background

During the last 3 decades, the Government of Nepal has attempted to eliminate malaria by improving the coverage and quality of indoor residual spraying (IRS), long-lasting insecticide-treated nets (LLIN), rapid malaria diagnosis (RDTs), and artemisinin-based combination therapy (ACT). Consequently, suspected and confirmed malaria cases, as well as their case severity, have been decreasing [1]. Nepal has achieved the Millennium Development Goals target on halting and beginning to reverse the incidence of malaria [1].

Malaria elimination may reduce human suffering and financial burden on households and foster economic growth through increased worker productivity [2, 3]. Therefore, the Government is implementing the Nepal malaria strategic plan (NMSP) 2014–2025 with a goal to eliminate the disease by 2025. The focus of the strategy is on improving quality of and access to early diagnosis and effective treatment of malaria and strengthening programmatic, technical and managerial capacities towards malaria elimination [4].

Beyond policy formulation, eliminating malaria will require a higher level of sustainable financing, cooperation among the health service providers, affordable and effective malaria medicines and commodities, and evidence-based decision making, among others [3]. Two factors make these changes and malaria elimination difficult to achieve in the case of Nepal. First, as in many countries, the government allocates annual budget based on national-level priorities and, to some extent, the burden of diseases. Until a few decades ago, Nepal had a high prevalence of communicable diseases, such as malaria, relative to non-communicable diseases (NCDs), such as hypertension and diabetes [5, 6]. As in many low and middle-income countries (LMICs), this has shifted in recent years in Nepal [6], with NCDs now accounting for 66% of all deaths and 48.5% of all premature deaths [7]. The priority of the government is likely to shift towards addressing the rising burden from NCDs. Timely and fine-grained data on allocation and spend by disease are unavailable. Nonetheless, available data suggest that total expenditure on malaria *decreased* from Nepalese rupees (NRs) 117.9 million (approximately US$1.03 million at current exchange rate) in fiscal year (FY) 2012–2013 to NRs 99.2 million in FY 2013–2014, and further to NRs 97.7 million in FY 2014–2015 [8].

Second, the burden of malaria is localized in certain geographic areas, and the new constitution assigns the management of basic healthcare services, such as prevention, diagnosis and treatment of malaria to local governments [9], which have limited capacity on prevention, diagnosis and treatment of malaria. The health posts, which are the first point of contact for patients, lack a sufficient number of staff designated for malaria. Although this may change after the current transitional period is over (the country is still institutionalizing the federal system which it adopted in 2017), generally, it has been well documented that the current human resource base of approximately 35,000 health workers is insufficient to provide adequate care to the current population of Nepal [9].

Against this background, the authors conducted a qualitative study with a capacity-building exercise embedded within it. The research component aimed to understand how policymakers and local actors perceive challenges in malaria elimination. For them to be able to articulate the challenges, however, an understanding of malaria elimination in the context of a broader health system in Nepal would be required. The capacity-building component, thus, involved providing that knowledge.

## Methods

### Overview of the health system in Nepal

The Ministry of Health and Population of the Government of Nepal designs health policies and allocates human and financial resources to deliver healthcare services throughout the country, including on malaria. Within the Ministry, the department of health services (DoHS) is responsible for delivering preventive, promotive, diagnostic, and curative health services. The DoHS currently has 7 divisions and 6 centres. The Epidemiology and Disease Control Division (EDCD) is responsible for design and implementation of malaria interventions. The Vector-Borne Disease Training and Research Centre (VBDTRC) provides basic and refresher training on malaria microscopy for laboratory personnel.

The Federal Government provides 4 types of grants to the provincial and local governments, including conditional grants for health and education [10]. Provincial governments also have their own sources of revenue, which they can allocate to the health sector within the provinces. Except in the case of a few large programmes of the government, such as the *Aama* programme (aimed at reducing child and maternal mortality), grants to local municipalities from Federal and provincial governments are not tied to specific programmes. Therefore, local municipalities have discretion on how to prioritize available funds among their competing programmes, including those on malaria prevention, diagnosis and treatment.

At the local level, health posts are the first institutional contact point for individuals seeking care, including on malaria. Health posts monitor and provide support for the community-based activities of primary healthcare outreach clinics (PHC-ORCs) and the activities of female community health volunteers (FCHVs). The health posts refer cases to the primary healthcare centres (PHCCs) which in turn refer them to district hospitals. district public health office (DPHO) [sometimes referred to as the District Health Office (DHO)] coordinates the local health offices and is responsible for effective delivery of healthcare services, including malaria care services, and the management and administration of the district health system.

### Epidemiology of malaria in Nepal

The epidemiology of malaria in Nepal has been documented [11, 12]. Briefly, based on annual parasite incidence (API), the government has classified 75 districts in the former administrative structure as high-risk districts (API ≥ 1), moderate-risk districts (API = 0.5–1), or low-risk districts (API = 0–0.5). In 2012, the latest year for which data were available at the time of writing, 13 districts were high-risk, 18 were moderate-risk, 26 were low-risk, and the remaining 8 were considered no-risk [4] (see Fig. 1 in Dhimal et al. for a map of Nepal showing districts by their risk status [12]).

Malaria transmission occurs year-round in Nepal, peaking in July during the seasonal monsoon. Risk groups include ethnic minorities, the poor, young individuals, mobile populations, and people living near the Indian border. Although the government provides diagnosis and treatment for malaria free of cost in government health facilities, in reality the lack of medicines and human resources in these facility, high travel costs, and socio-cultural factors discourage the poor and vulnerable individuals from seeking and utilizing care [13].

In recent years, two developments have been observed in the epidemiology of malaria in Nepal. First, malaria vectors have shifted to higher altitudes due to climatic and environmental changes [11]. Second, there has been a marked rise in the number of imported cases, particularly that of the *Plasmodium falciparum* [1].

### Study setting

Apart from a brainstorming session at the central level, the authors conducted the study in three endemic districts in far western Nepal: Kanchanpur, Kailali and Dadeldhura. The three districts are located within the *Sudurpaschim* (meaning: far west) province, which, together with the Karnali province, accounts for 80% of the high-risk burden of malaria [1]. In the three districts, the slide positivity, or the prevalence rate, ranged between 0.7 and 2.9%, and the treatment rate was between 90.8 and 100% at the time of this study (see Table 1).

**Table 1:** Basic malaria indicators in the study districts

Indicator	Kanchanpur	Kailali	Dadeldhura
Blood slides tested	13,410	10,187	727
Positive cases (% of total sample collected)	87 (0.7)	295 (2.9)	14 (1.9)
Number of positive cases treated (% of slide positivity)	85 (97.7)	268 (90.8)	14 (100)
*Plasmodium vivax*			
Indigenous	34	121	5
Imported	48	48	9
*Plasmodium falciparum*			
Indigenous	1	7	0
Imported	5	18	0

District Health Offices of respective district, 2016. This table shows the number of cases. For example, blood samples were tested among 13,410 individuals in Kanchanpur district. Of them, 87 (approximately 0.7%) were found to be positive

### Capacity-building exercise

The capacity-building component of this study had three key steps: (1) brainstorming session among policy makers and implementers at national and local level; (2) providing training to community members and local healthcare providers; and, (3) assessing the understanding of the system’s thinking and financing approaches for malaria elimination among key stakeholders at the local level.

### Brainstorming on elimination of malaria and health system thinking

The authors conducted a brainstorming workshop, first at central level, then at community level, to develop content for the training to be provided at local level. These brainstorming sessions differed from focus group discussions. Specifically, the authors prepared a set of issues to share with the participants and sought their comments on each issue, moving forward in a linear fashion. SA acted as facilitator, rather than moderator.

The workshop at central level focused on the demand for and supply of malaria care services at community level. SA discussed the 6 building blocks of the health system [14]. The brainstorming session was guided by the ‘Policy Delphi’ method where all participants presented their options and views, in this case, on health system- and economic-related challenges to making local care delivery on malaria more effective [15, 16]. The participants also discussed local resource mobilization, various financing options and coordination between local level agencies.

To identify participants for the central-level brainstorming exercise, the authors used purposive sampling. The key inclusion criteria were: (1) policymakers working in the field of malaria control for at least 5 years; (2) senior government officers with significant knowledge and extensive work experience in malaria control; and, (3) vector or malaria control officers or experts with more than 2 years at the local level. Ten experts were selected based on these criteria.

At community level, the brainstorming exercises centred on the supply of and demand for malaria care services at community level. These sessions were conducted in 4 locations (one per district, except in Kanchanpur where there were 2 locations). The participants included health post staff, female community health volunteers, laboratory technicians, and current and past members of health post management committee. The research team went to the health posts and requested staff to contact these individuals. The participants were drawn from the same districts where the training was conducted. The number of participants ranged from 5 to 9.

Finally, an additional session was conducted at the district level. The participants in this session included the chief district officer, the local development officer, the water and hygiene and sanitation officer, representatives from the zonal hospital and other vector control programme officers at DPHO from the three districts. This event was conducted in Kanchanpur district. The DPHO of Kanchanpur district wrote an invitation letter to participants on the research team’s behalf. A total of 11 participants from three districts took part in this session.

### Training on health system thinking and financing approaches

Following the brainstorming sessions, the authors developed a training manual consisting of 8 modules (see Box 1) related to malaria prevention, treatment and programme financing. These modules were based on existing evidence of the critical role that various aspects of the health system play in service delivery in low-income settings [14]. For example, availability of medicines, human resources and proper information flow are essential elements to determine the utilization rate of health care [17–20]. Similarly, provider behaviour is important in determining user satisfaction [21]. Access to healthcare services, resources and facilities determine the effectiveness of the system. Availability of human resources, diagnostic testing, quality of medicine, quality of preventive services, patient adherence, and individual benefit determine effective coverage of malaria intervention [2, 19, 20, 22]. Health system inefficiency is directly related to quality of care, quality of drugs, and behaviour of providers [21–23]. Two additional modules specific to malaria were added: one on the health system approach and one on the epidemiology and economics of malaria.

The training was conducted in June 2016. Five health institutions (3 from Kanchanpur, 1 from Kailali and 1 from Dadeldhura) were randomly selected from the list of health institutions in endemic areas of those districts. The institutions included 3 health posts and 2 primary health care centers (PHCCs). Participants in the training and workshop were in-charge of these institutions, health staff, FCHVs, members of management committees (of health posts and PHCCs), service recipients, civil society, and local people. In each health institution, the head staff were asked to identify individuals in their locality based on their possible role in prevention and treatment of malaria eradication. These individuals came from all walks of life, including teachers and journalists. A total of 17 participants took part in training (3 each from the 3 health posts and 4 each from the 2 PHCCs).

One of the main reasons for including training in this study was to involve both health and non-health professionals. Nepal has an integrated health system, in which non-health professionals are often engaged in the budgeting process. It would be important to engage them and build their knowledge. Some participants had a sophisticated knowledge of malaria elimination in Nepal, while others had little or no knowledge. The training was intended to help bridge this gap.

#### Box 1: Outline of the training manual

*Health system approach to control malaria* This module included an introduction to system thinking and recent developments in system thinking for malaria.

*Epidemiology and economics of malaria* This module included a discussion of epidemiological features of malaria and the economics of its prevention and eradication.

*Governance and leadership* This module covered strategic policy frameworks for effective oversight, coalition building, accountability, transparency, regulations, incentives, and system design.

*Health workforce* This module covered approaches to developing a sufficient, responsive, fair, and efficient health work force given available resources and circumstances.

*Health financing* This module covered issues such as raising adequate funds for health in ways that ensure people can use necessary services without facing impoverishment from out-of-pocket costs.

*Health technologies* This module covered medical products, vaccines, diagnostics, and other technologies of assured quality, safety, efficacy, and cost-effectiveness.

*Health information* This module discussed approaching to ensuring the production, analysis, dissemination, and use of reliable and timely information on health determinants, health systems performance, and health status.

*Service delivery* including effective, safe, and quality personal and non-personal health interventions that are provided to those in need, when and where needed (including infrastructure), with a minimal waste of resources.

### Assessment of the gaps on current initiatives for malaria elimination

Following training, the authors conducted focus group discussions (FGDs) (2 in Kanchanpur district and one each in the other 2 districts) and key informant interviews (KIIs) to assess local stakeholder (individuals who participated in the FGDs) perception of key gaps in service utilization and delivery in relation to malaria elimination. The opportunity was taken to assess participants’ understanding of the system approaches to malaria elimination.

The participants for the FGDs were selected and invited following the same procedure as was used for the training. A maximum of 12 participants per focus group allowed sufficient time for sharing opinions. The participants included those who had received training (2–5 individuals in each group) but the majority had not received training. The moderator (SA) introduced and highlighted the purpose of the meeting and encouraged participants to provide their honest views. The discussion took place in Nepali, the local language. FGDs lasted for up to an hour and the moderator stopped the session when he felt that the points were being repeated.

Participants for the KII were selected based on participation at the FGDs (2 per FGD, for a total of 8 individuals). In each FGD, the authors identified 2 individuals who participated most actively and asked them if they were willing to provide in-depth interviews.

Hand-written field notes provided an account what was heard, seen, or experienced during the FGDs and KIIs. These notes, once collated, were compared against audio recordings to ensure important points were not missed in the written notes. The notes were translated and common themes were identified through discussion between SA, VS and AT on the basis of their independent initial analysis (each author prepared a menu of themes). The analysis was an iterative process, whereby themes were continuously generated, revised and re-examined. This method followed the approach used in Adhikari et al. [24].

## Results

### General perceptions of malaria trends in three districts

During FGDs and KIIs, respondents said that “…malaria has drastically decreased [from] previous years”; that many cases were “imported from India” (i.e., detected in individuals who had travelled to India); “5 imported cases from India were identified… mostly the cases are imported”; that the prevalence rate was low “within *Jestha* to *Shrawan* (June-July) among the 300 tested only 3 cases were found to be malaria positive”; and that the poor and disadvantaged groups were more likely to be affected than their rich and advantaged counterparts (“…communities like Kamaiyas, landless labours, poor and marginalized are prone to malaria”).

Despite the noticeable decline in malaria incidence in Nepal, the participants also expressed fear that efforts on malaria prevention had been substantially reduced (“as the malaria cases are falling the attention towards prevention is falling by 90% of previous efforts”) which could reduce the chance of malaria elimination (“we cannot guarantee the total elimination of malaria with present efforts and manpower if any epidemic breaks up”).

In the rest of this section, key demand and supply factors that local stakeholders (participants at the FGDs and the KIIs conducted in the 3 districts) perceive as strengths and challenges to malaria elimination in Nepal are discussed. A summary of the following discussion is provided in Table 2 for easy reference.

**Table 2:** Key gaps between demand and supply of malaria control interventions and program components

Areas	Demand	Supply	Local perception of the gap
LLIN	There is a high demand for LLIN at community level. Even existing LLIN need replacement as many may have worn out. The willingness to pay for LLINs is low, however, especially among the disadvantaged groups	The last distribution of the LLINs by the district authorities was 4 years before. Currently, only high-risk groups, such as infants and pregnant mothers in high-risk geographic areas them. The government attempted to provide LLINs to disadvantaged groups a few years ago with limited success	There is an excess demand for LLIN
IRS	There is a high demand for IRS at the community level, especially in high-risk areas, such as the ones near the forest belt and brick factories In suburban areas, households are buying IRS themselves to spray at community level as they suspect the quality of IRS sprayed by the government	Supply of IRS is low at community level and the district public health offices have targeted specific areas to spray each year (free of cost)	There is an excess demand for IRS
Diagnosis and treatment	There is a high demand for the diagnosis and treatment of malaria, mainly due to increased awareness among the population	Many health facilities are equipped with basic diagnosis and treatment services. However, microscopy services are available only in a few health facilities, as are laboratory chemicals	There is a lack of microscopy services for testing blood samples in health facilities
Human resource	The communities seek qualified health workers in the public health facilities and either seek alternatives (i.e., the private providers) or forego diagnosis and treatment when they are not available	There exists a shortage of skilled and trained health workers in all health facilities. The existing human resources, including the few lab technicians, health assistants, have limited training on malaria diagnosis and treatment. The female community health volunteers also lack malaria-specific knowledge	There are insufficient number of health workers and in cases where they are present, they are insufficiently trained on the diagnosis and treatment of malaria

#### Demand: strengths and challenges

The strengths and challenges reported by local stakeholders related to their understanding of the significance of LLINs, IRS, diagnosis and treatment, and community efforts.

#### LLIN

Stakeholders suggested that demand for LLINs was increasing at community level. The respondents expressed that LLINs were better than the usual mosquito nets available in the market, and that individuals in their communities had realized the usefulness of LLINs to prevent malaria.

*“Nowadays even the poorest people in the society use the mosquito nets; they use it even after sewing the holes in it”.*

In one instance, a local person paid for LLINs on behalf of a poorer individual who could not afford the price (NRs 100).

#### Insecticide residual spray (IRS)

FGDs at community level revealed that the DPHO has been conducting IRS in selected high-risk pockets. Most of the households were covered in those pockets. However, one community leader pointed to the decreasing efficacy of these sprays

*“These days the spray is not as effective to kill the mosquito as it used to be, we see a lot of mosquitoes even after the spray”*.

Another individual suggested that, in certain areas, the spraying was not as intensive as it should be

*“a few risky pockets such as the communities living nearby forest belts and those living nearby brick industry require intensive IRS which is yet to be met by the government authority”*.

There seems to be a large demand for IRS at the community level. Those living in the suburbs of the district headquarters in one district were spraying individually (i.e., with their own resources).

#### Diagnosis and treatment

FGDs at community level showed that people were aware of the diagnosis and treatment of malaria available in government facilities, but were wary of the quality of the care they would receive, in particular the timeliness. As a result, they often went to a private facility instead.

“*If an individual in community gets fever, we directly go to private clinic because we get quicker access to the services”.*

The respondents also pointed to specific challenges in accessing a public facility, such as the lack of medicines, qualified health professionals and long wait times.

*“medicines are not available in public health facilities, … very less frequently they do have enough medicines for us, quite often, human resources are not available while accessing facility, and diagnosis also takes time as compared to private facility”.*

Nonetheless, treatment-seeking behaviour in general seems to be improving with individuals consulting the health facility as soon as they suspect a fever.

*“… people these days do not delay in consulting health facility (public or private) whenever they get any sort of fever…”).*

### Household and community efforts

FGD at community level explained that communities had taken initiatives to prevent and control malaria. At household level, individuals took measures such as wearing long-sleeved clothes to prevent mosquito bites, reducing outdoor activities, using sieved doors, using dung cake smoke to repel mosquitoes, and removing open-stored waters from around the house.

The communities collectively engaged in the removal of open-stored water bodies (such as ponds); the removal of rubbish and bushes; school health programmes to promote malaria prevention strategies; community awareness about externality and spill-over effect of malaria control initiatives; and, awareness about “importing” malaria from travelling.

### Supply: strengths and challenges

The supply strengths and challenges participants identified can be categorized into 6 broad areas: governance, finance, medicine and technology, service delivery, human resources, and information.

### Governance

Respondents’ opinions about the governance of programmes on malaria prevention, diagnosis and treatment varied. Respondents provided examples of instances where local healthcare providers had allocated additional resources.

*“……the management committee has contributed a lab assistant locally which is vital for diagnosis and treatment of malaria…”*.

However, most of the opinions expressed concerns, as the following statements show.

*“[The] local municipality is organizing public accounting sessions annually regularly, however the participation of local public is not as much as is generally expected”.*

*“The reason for reduction in malaria may be attributed to Roll*-*back Malaria … in past years.”*

*“As malaria cases are falling the attention towards prevention is falling to 90% of previous efforts.”*

*“…Mostly the cases are imported and local authority pays less attention on it.”*

*“Now no more quarterly meetings held for malaria prevention and slide collection campaigns…”*

Several comments had to do more with shortcomings in the system generally than with malaria prevention, diagnosis or treatment in particular, as the following statements show.

*“… Health is not on the top priority list of the Municipality.”*

*“… [Local] management committee though monitors the health facility staff time to time, we have virtually less power to exercise when it comes to correcting staff”.*

### Finance

Based on FGDs, public primary facilities spend 5–10% of their total budget on malaria-related activities.

“*we spend 5*–*10% budget in some ways for malaria*”.

In some cases, local government bodies seem to be covering the salaries of lab technician and nursing staff.

“*…lab’s expenditure and salary of the lab technician is managed by health post management committee.”*

*“The development committee of Municipality is providing … salary amount to me*.”

However, out-of-pocket expenses for malaria prevention remain high for individuals, even in areas where the health facilities should be providing mosquito nets to them.

*“…Some of them has been using even by purchasing other mosquito nets from market.”*

Costs may also be high because of individuals needing to go to private facilities.

*“People… go to private clinics and drugstores for diagnosis and treatment.”*

### Medicines and technology

The availability of medicine for malaria in public facilities is uneven. A number of facilities reported rationing because of shortages:

*“There is also shortage of RTD testing kits so …Only those cases of fever with some observed symptoms are tested for RTD”*

*“We cannot cover whole community for IRS, therefore, we are covering only high risk and pocket areas. We do have limited amount of IRS available from the district”*

*“This year LLIN nets were distributed only to ANCs.”*

*“Sadly there is high demand of nets and sprays but we have not been able to provide them any.”*

*“It has been more than 3* *years the last time government distributed the LLIN. We would like to request the concerned authority to distribute LLIN again as we have realized that these nets are quite useful”.*

A few facilities reported having sufficient availability:

“*[There] is no shortage of medicines for malaria in our stores”*.

*“Almost all health facilities here have basic diagnosis and treatment facility available.”*

### Service delivery

The respondents said that the quality of service received from public facilities was poorer than the one available from private ones. Therefore, those who afforded preferred to visit the private providers.

“*People… generally, go to private clinics and drugstores for diagnosis and treatment”; “nowadays, more people seek services from private sectors than from us.”*

One key advantage the private providers had relative to the public ones were their flexible hours *“…the private sectors flexible time …attract much more patients than in public health facilities…”*

Public providers, on the other hand, had concerns about whether the individuals themselves were using the LLINs properly.

“*LLINs were distributed but whether they are using properly or not is another story…”*.

### Human resources

The respondents reported several problems ranging from the absence of doctors due to their frequent transfer by the government, insufficient laboratory facilities, and lack of fresher trainings for the health outreach workers.

*“…availability of doctor is the problem, they are frequently transferred..”; “During their (doctors) absence … people face real problems of health”*

*“There is huge work load in lab. …”*

“*The FCHVs also need training programmes related to malaria every 1*-*2* *years because their role is vital in malaria awareness”*.

### Information

There was generally consensus among the respondents that an increase in awareness was a major factor driving recent declines in malaria cases.

“*The decrease in malaria case is solely due to increase in public awareness”*.

The providers knew that a significant part of their responsibility was to educate communities on the prevention of malaria.

“*The role of health organizations is basically to educate and make aware people regarding malaria).*

Many providers indicated that they organized public awareness campaigns.

“*We organize various programmes among which malaria also falls…”*

although these events were often not exclusively about malaria.

*“… there are public hearings from time to time. But …does not focus only on malaria…”*).

## Discussion

As summarized in Table 2, local participants in this study reported gaps in human resources, diagnosis and treatment, the provision of LLINs, and the conducting of IRS in the study area in Nepal. The findings are consistent with studies done elsewhere where unmet demand has been observed [20, 21]. In terms of diagnosis and treatment, the key gap is the lack of microscopic services. This finding is also consistent with previous studies [13, 22, 25, 26].

The primary healthcare centres and health posts in the study area, which are the first point of contact for service seekers, reported that they spend between 5 and 10% of their total budget on malaria control activities. However, there exist significant out-of-pocket expenditures for malaria diagnosis and treatment as many individuals visit private service providers due to lack of diagnosis facilities, the poor quality of care, and in many instances an absence of qualified human resources, in public health centres. The absence of skilled human resources in health was one of the frequently referenced problems by participants. Currently there are no health staff who are designated specifically for malaria in the health posts. If not addressed, this will be a major constraint for malaria elimination in Nepal; adequate human resources have been found to be central to such efforts around the world [27].

This study has a number of limitations. The authors relied heavily on local health facilities to identify respondents for the FGDs. Given this non-random sampling, the messages quoted may not be representative of the overall population in the districts, and are vulnerable to desirability bias, as the training preceded the FGDs and some of the FGD participants had attended training as well. Given these limitations, the findings should be considered suggestive. In particular, the difference between what stakeholders perceive as necessary and what is available should be understood as a *local perception* of the gap in services. For effective policy design, a further corroboration and quantification of the gaps by locality, perhaps through a larger survey, would be required.

The approach used, however, may be applicable to other settings that are approaching malaria elimination and where the changing disease burden is likely to shift a central government’s efforts away from localized diseases, such as malaria. First, it is possible to capitalize on local expertise in order to identify gaps in malaria elimination at a sub-national level by undertaking a capacity-building exercise within a study. This would be an appropriate approach when sub-national quantitative data are not available, such as is the case in Nepal. Second, where management of localized conditions such as malaria may shift to local governments, the study itself may provide an opportunity to raise awareness and expertise of local stakeholders. In this study, the training on the system’s approach to policy analysis (see Box 1) likely increased the knowledge of malaria management, which local policymakers can use for conditions other than malaria.

## Conclusion

With the shifting burden of the disease toward non-communicable conditions and the localized nature of malaria prevalence in many countries around the world, the priorities of central government are likely to change, affecting financing for malaria elimination. In the context of Nepal, it was possible to simultaneously strengthen local capacity while understanding local perceptions of challenges to malaria elimination. Locals in three endemic districts of Nepal perceive that there are significant gaps in human resources, diagnosis and treatment, the provision of LLINs and the conducting of IRS.

## Data Availability

All data generated or analysed during this study are included in this published article

## Abbreviations

ACT: artemisinin-based combination therapy
API: annual parasite incidence
DHO: district health office
DPHO: district public health office
DoHS: department of health services
EDCD: The Epidemiology and Disease Control Division (EDCD
IRS: indoor residual spraying
LLIN: long-lasting insecticide-treated net
NCD: non-communicable diseases
NMSP: Nepal Malaria Strategic Plan
PHCC: Primary Health Care Center
RDT: rapid diagnostic test
VBDTRC: Vector-Borne Disease Training and Research Center
